# Rapid high throughput template preparation (rHTTP) method: a novel cost effective method of direct PCR for a wide range of plants

**DOI:** 10.1186/s12896-019-0560-4

**Published:** 2019-10-26

**Authors:** Prassan Choudhary, Sudipta Das, Hillol Chakdar, Arjun Singh, Sanjay Kumar Goswami, Anil Kumar Saxena

**Affiliations:** ICAR-National Bureau of Agriculturally Important Microorganisms (NBAIM), Mau, Uttar Pradesh 275103 India

**Keywords:** Plant- DNA- high throughput- PCR-SSR

## Abstract

**Background:**

Conventional plant DNA isolation methods are complex, time consuming and require technical expertise. These limitations were overcome using the DNA isolation kits which, however significantly add to the research costs. Hence the present study was aimed to develop a high throughput, rapid and inexpensive method of PCR ready DNA template preparation from plant materials.

**Methods:**

Concentration of SDS in lysis buffer, amount of starting material, period and temperature for lysis were optimized for obtaining PCR ready templates from plant materials. The method was tested using RAPD and ITS specific primers for different plant species like rice, wheat, mustard, pea, soybean, pigeonpea, tomato, maize, march lilly, bougainvillea, Indian blanket flower, nerium, petunia, purple pirouette petunia, moses-in-the-cradle, golden cane palm, duranta, periwinkle, chrysanthemum and two xerophytes viz. *Dipterygium glaucum* and *Crotaleria burhia*. SSR markers RM18398 and RM26108 showed successful amplification in rice varieties Improved Pusa Basmati 1 and KS Dev 12. The effectiveness of the method was tested using fresh as well as 1 year old tissues. The storability of the lysate was also tested.

**Results:**

In this report, we developed a novel method called rapid high throughput template preparation (rHTTP) method to prepare PCR ready DNA templates. Most striking feature of this technique is that it can be done anywhere where water can be boiled by any means. Using rHTTP method, PCR ready templates can be prepared in just 10 min. Robust and reproducible amplification for all the test plants were recorded with RAPD, plant ITS primers and SSR markers following this method. rHTTP methods works well for both fresh as well as old plant tissues. The lysates had a shelf life of 1 month when stored at 4 °C and 3 days when stored at room temperature.

**Conclusions:**

rHTTP method has several advantages over the other protocols like ease of execution, no requirement of tissue grinding/liquid nitrogen/hazardous chemicals and above all, equally effective for both fresh and old samples. Using this method, costs per prep comes down ~ 10–50 times as compared to most commercial kits. This method can be used for on-field experiments like molecular diagnostics, varietal identification etc.

## Background

DNA extraction is one of the most important and critical steps of a molecular biology laboratory. The conventional methods of DNA isolation involved multiple steps to rupture the cells and purify nucleic acids from the complex mixture of macromolecules using hazardous chemicals like phenol, chloroform etc. Such methods were also considerably lengthy and impeding the speed of work when DNA had to be isolated from a large number of samples. In the early 1990s, efforts were being made to simplify the cumbersome methods of obtaining good quality plant DNA which could be useful for downstream processing [1–4]. Rapid DNA fingerprinting techniques were also developed necessitating the scientists to work towards reducing the time and effort required for DNA isolation [4]. Varma et al. (2007) reviewed the various aspects of plant DNA extraction complexities and concluded that the chemical heterogeneity and diversity of plant systems rendered it very difficult for a single method to be employed for isolating pure, restrictable plant genomic DNA [5–11]. In 1993, Wang et al. reported a rapid NaOH based method for DNA template preparation for direct PCR [12]. The methodology was reported to be unsuitable for amplification of products more than 600 bp and consequently, Steiner et al. developed a new buffer (ROSE buffer) composition for DNA extraction [13]. Gradually, a number of modifications of the NaOH based methods have been reported by a number of researchers [14–17]. Apart from NaOH, sucrose has also been used for rapid extraction of DNA from plant tissues [18]. Few “touch and go” methods of direct PCR have also been reported [18, 19]. Research in this regard got a flip with the development of commercial kits which mostly rely on spin columns that capture the nucleic acids from cleared homogenates. However, the use of commercial kits attracts a considerable financial burden for research works. Despite the continuous development of a number of rapid DNA isolation methodologies since last 25 years, their use has been very limited. Satya et al. (2013) emphasized that reliability, wide application, storability of the extracts or even sometimes simple mental bias might have been the reasons behind their poor popularity among the scientific community.

On comparison of few reported DNA template preparation methods, it was realized that most of the so called “rapid methods” still require liquid nitrogen or lyophilized tissue, essentially grinding of the tissue and use of instruments like centrifuge or vortex. These operations reduce the sample processing efficiency as many of these had to be done individually or separately for each sample. Hence, these so called “rapid methods” seemed not to be very rapid when we need to process hundreds of samples together. However, such methodologies have potential to be used for large scale genotyping, DNA barcoding, molecular breeding and even in molecular diagnostics. In a recent report, Wang et al. (2016) conclusively demonstrated the use of rapid DNA template preparation in loop-mediated isothermal amplification (LAMP) for field detection of transgenic lines of rice in China.

Therefore, there was a need to develop a really rapid, simple method which can be used to yield PCR ready templates from a wide range of plant species and simultaneously cheaper than the commercially available kits. In this study, we report a novel, rapid and inexpensive method to prepare PCR ready template from a variety of plants and showed its usefulness in molecular biology experiments.

## Results

### Development and optimization of the method

The results on optimization of SDS concentration in lysis buffer revealed that highest DNA yield was achieved when 1% SDS was used (Table 1). The concentration of SDS did not specifically influence the purity of DNA. For further optimization studies, 1% SDS was used for lysis. Among the two different amounts of tissue used for lysis, the results on DNA yield was significantly better when two leaf discs (~ 3.2 mg) were used as a start material for lysis (Table 1). Among the two time periods (10 or 20 min) used for lysis,10 min was finally selected as the DNA yield was higher as compared to 20 min of lysis (Table 1). Hence, the optimized protocol for DNA template preparation included lysis of two discs (~ 3.2 mg) of leaf tissue in 100 μl of 1% SDS and heated for 10 min at 99 °C in a thermal cycler.

**Table 1:** DNA quality and yield in lysates prepared with different concentrations of SDS, different amount of tissue and different time duration

Parameters		DNA Purity (A_260_/A_280_)	DNA Concentration (ng/μl) ^a^
SDS conc. (%)	0.5	1.03	451.67 ± 8.82
	1.0	1.01	768.33 ± 6.01
	1.5	1.03	376.67 ± 4.41
	2.0	1.04	252.17 ± 1.48
	2.5	1.03	438.33 ± 14.24
	3.0	1.04	261.17 ± 21.17
	3.5	1.05	218.33 ± 3.33
	4.0	1.06	260.17 ± 3.06
	4.5	1.05	326.67 ± 1.67
	5.0	1.04	446.67 ± 14.81
Amount of tissue	One disc (~ 1.6 mg)	1.06	420.00 ± 10.08
	Two disc(~ 3.2 mg)	1.04	760.00 ± 6.75
Time durations	10 min	1.08	876.67 ± 37.12
	20 min	1.05	808.33 ± 6.01

^a^DNA concentration is represented as Mean ± SE

**Fig. 1:**
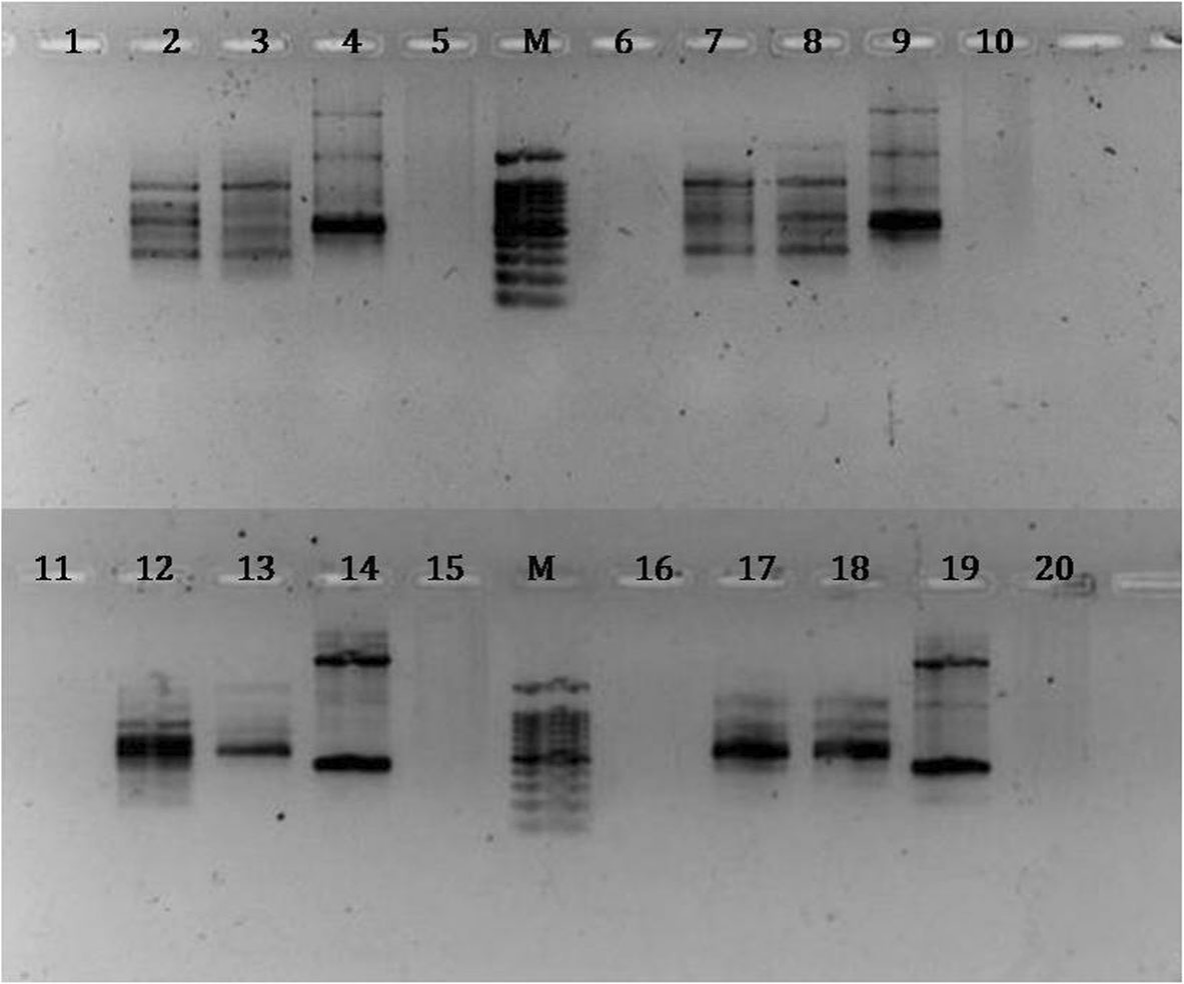
OPB6 (lane 1 to 10) and OPB7 (lane 11 to 20) primers showed good amplification results using10X and 20X dilutions of the crude template DNA lysate. Lane 1:Crude lysate (Cr) -without 1% BSA (BSA^−^); Lane 2: 10 times diluted (10X) lysate-BSA^−^; Lane 3: 20times diluted (20X) lysate-BSA^−^; Lane 4:Bacterial DNA (Bac)-BSA^−^; Lane 5: Bac- with 0.1% SDS-BSA^−^; M: 100 bp marker (Promega); Lane 6: Cr-with 1% BSA (BSA^+^); Lane 7: 10X lysate -BSA^+^; Lane 8: 20X lysate-BSA^+^; Lane 9: Bac-BSA^+^; Lane 10: Bac- with 0.1% SDS-BSA^+^; Lane 11: Cr-BSA^−^; Lane 12: 10X lysate-BSA^−^; Lane13:20X lysate-BSA^−^; Lane 14: Bac-BSA^−^; Lane 15: Bac- with 0.1% SDS-BSA^−^; M: 100 bp marker (Promega); Lane 16: Cr-BSA^+^; Lane 17: 10X lysate-BSA^+^; Lane 18: 20X lysate-BSA^+^; Lane 19: Bac-BSA^+^; Lane 20: Bac- with 0.1% SDS- BSA^+^

The success of the DNA template was judged by PCR amplification using RAPD primers (OPB06 and OPB07). An aliquot of 2.5 μl of crude lysate, 10X and 20 X diluted lysate were used as a template for two series of amplification reactions with or without BSA in PCR reactions for two different reaction volumes viz. 20 μl and 40 μl. No amplification was achieved with the crude lysate in presence or absence of BSA (Fig. 1). Likewise the bacterial DNA dissolved in 0.1% SDS did not yield any amplification product. Both 10X and 20X diluted lysate yield amplified products with no significant differences in the amplification profile both in presence or absence of BSA (Fig. 1). Therefore, 20X diluted lysate was used for further validation of our protocol for DNA template preparation followed by PCR amplification with no addition of BSA in the reaction mixture.

### Effectiveness of the optimized method

**Fig. 2:**
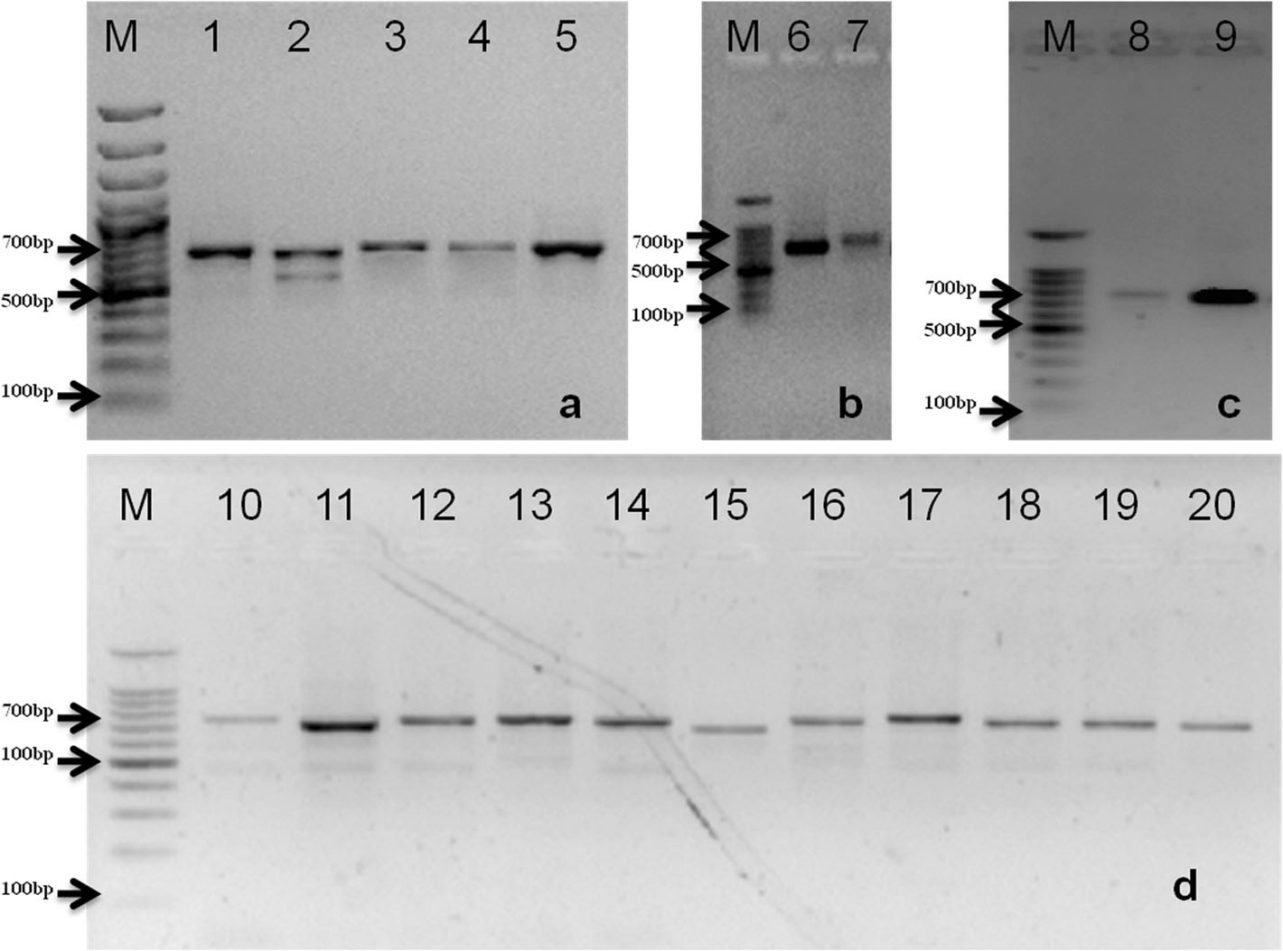
**a** Universal plant ITS primers depicted excellent resolution with the plant species tested. M: 100 bp marker (Promega); Lane 1: Mustard; Lane 2: Soybean; Lane 3: Pigeonpea; Lane 4: Wheat; Lane 5: Pea. **b** M: 100 bp marker (Promega); Lane 6: Tomato; Lane 7: Maize. **c** 100 bp marker (Promega); Lane 8: *Dipterygium glaucum*; Lane 9: *Crotaleria burhia*. **d** M: 100 bp marker (Promega); Lane 10:March Lilly; Lane 11: Bougainvillea; Lane 12: Indian blanket flower; Lane 13: Nerium; Lane 14: Petunia; Lane 15: Purple pirouette petunia; Lane 16: Moses-in-the-cradle; Lane 17: Golden cane palm; Lane 18: Duranta; Lane 19: Periwinkle; Lane 20: Chrysanthemum

**Fig. 3:**
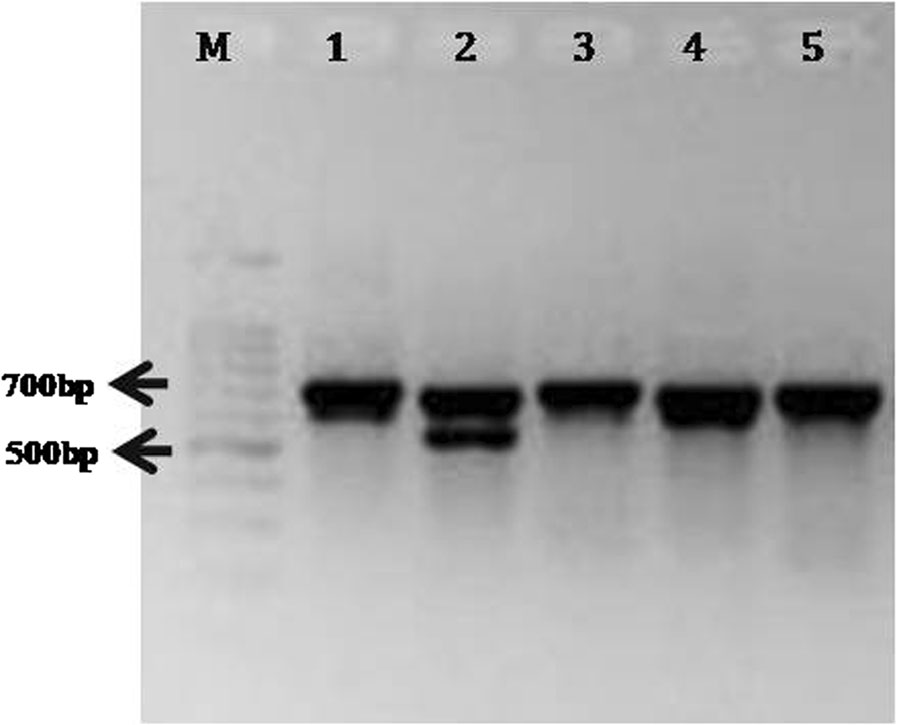
ITS amplification using 30 days old template DNA of the different plant species stored at − 20 °C. M: 100 bp marker (Promega); Lane 1: Mustard; Lane 2: Soybean; Lane 3: Pigeonpea; Lane 4: Wheat; Lane 5: Pea

The usefulness of this rHTTP method was validated for 11 different rice varieties and DNA template obtained resulted in robust RAPD profile upon PCR amplification (Additional file 1: Fig. S1). The leaf lysates of wheat, pigeonpea, mustard, soybean, pea, tomato, maize, march lilly, bougainvillea, Indian blanket flower, nerium, petunia, purple pirouette petunia, moses-in-the-cradle, golden cane palm, duranta, periwinkle, chrysanthemum, *Dipterygium glaucum* and *Crotaleria burhia* yielded a ~ 700 bp amplicon with universal plant specific primers for internal transcribed spacer (ITS) region (Fig. 2 a-d). The rHTTP method was equally effective for old tissues as lysis of 2 months old rice leaves of four different varieties yielded a DNA template that gave strong amplification using RAPD primer OPB07 (Additional file 2: Fig. S2). Figure 3 showed the results of PCR using templates stored at − 20 °C for 30 days. DNA templates prepared following rHTTP method showed intense bands of ITS regions of the tested crop plants. This indicated that the DNA template preparation using rHTTP method was stable over 1 month. In another experiment, the lysates were kept at room temperature (~ 20–22 °C) and checked for PCR amplification after 3 days. Additional file 3: Fig. S3 showed that even after storage of 3 days at room temperature, enough DNA remained in the lysate for PCR amplification.

**Fig. 4:**
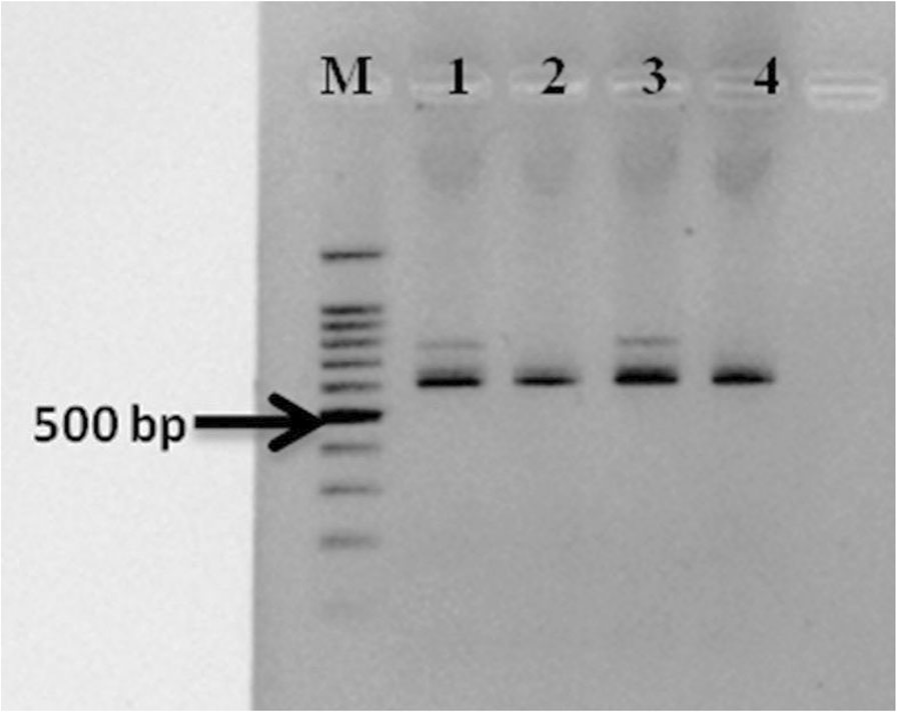
Amplification of ITS region of rice (varieties Improved Pusa Basmati 1 and KS Dev 12) using templates prepared in boiling water for 10 and 20 min. M: 100 bp marker (Promega); Lane 1: Improved Pusa Basmati 1_10 min; Lane 2: KS Dev 12_10 min; Lane 3: Improved Pusa Basmati 1_20 min; Lane 4: KS Dev 12_20min

The validated rHTTP protocol yielded a strong ITS amplicon even when boiling water (100–102 °C) was used for 10 and 20 min instead of heating the lysis mixture in thermal cycler. Strong ITS amplification was observed for both the time periods (Fig. 4).

**Fig. 5:**
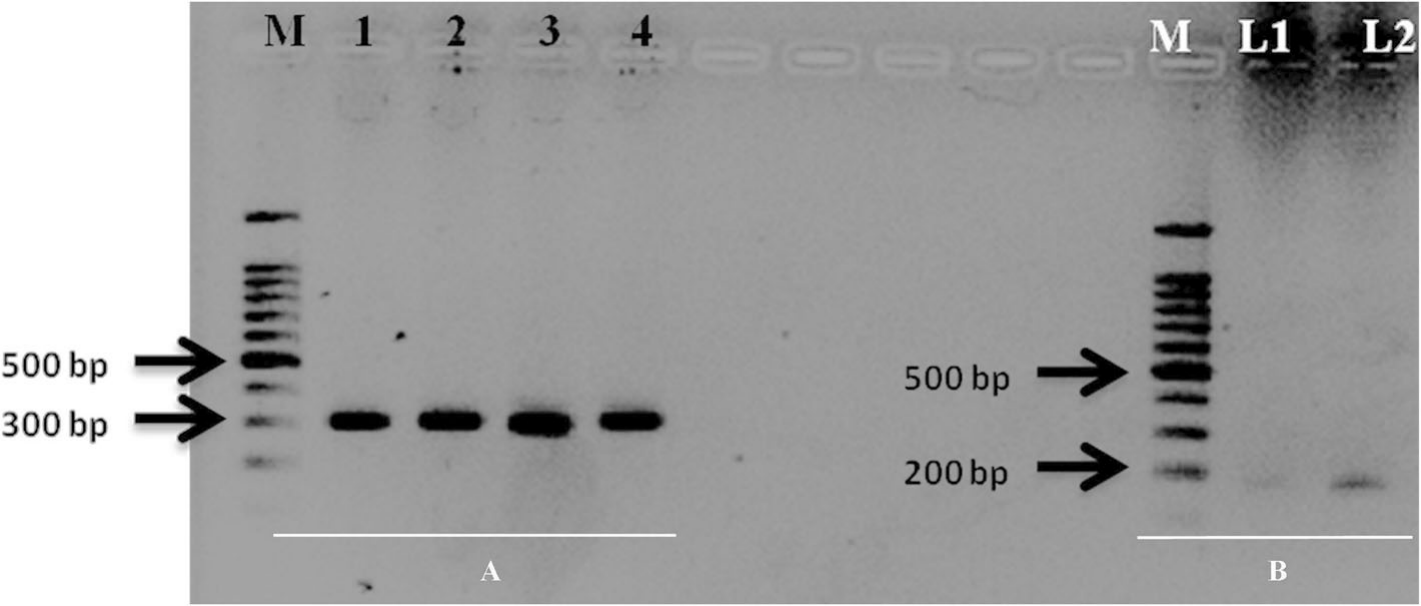
Amplification of SSR markers RM18398 (Panel A) and RM26108 (Panel B) showed successful amplification in rice varieties. Panel A: M: 100 bp marker (Promega); Lane 1: Pusa basmati 1121; Lane 2: Improved Pusa Basmati 1; Lane 3: KS Dev 12; Lane 4: MTU 5204. Panel B: M: 100 bp marker (Promega); L1: Improved Pusa Basmati 1; L2: KS Dev 12

SSR markers RM18398 (305 bp) and RM26108 (195 bp) showed good amplification with template prepared by rHTTP method as shown in Fig. 5a-b.

**Fig. 6:**
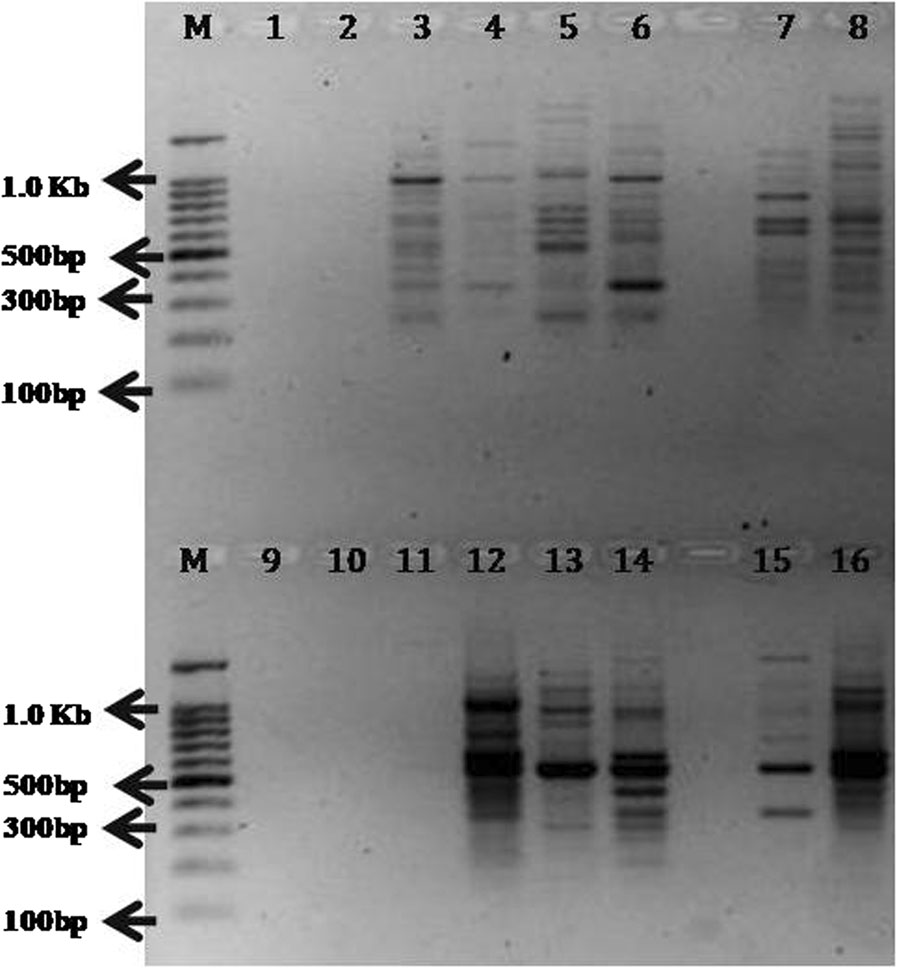
Comparative analysis of RAPD profiles of rHTTP and methods reported by Wang et al. (2016), Wang et al. (1993) and Satya et al. (2013). TKM13 and HUR917 varieties of rice were used for this PCR assay. Lane 2 to 9: Primer OPB06; Lane 10 to 16: Primer OPB07. M: 100 bp marker (Promega); Lane 1: TKM 13_Wang 2016; Lane 2: HUR 917_Wang 2016; Lane 3: TKM 13_Wang 1993; Lane 4: HUR 917_Wang 1993; Lane 5: TKM 13_Satya 2013; Lane 6: HUR 917_Satya 2013; Lane 7: TKM 13_rHTTP method; Lane 8: HUR 917_rHTTP method; M: 100 bp marker (Promega); Lane 9: TKM 13_Wang 2016; Lane 10: HUR 917_Wang 2016; Lane 11: TKM 13_Wang 1993; Lane 12: HUR 917_Wang 1993; Lane 13: TKM 13_Satya 2013; Lane 14: HUR 917_Satya 2013; Lane 15: TKM 13_rHTTP method; Lane 16: HUR 917_rHTTP method

**Fig. 7:**
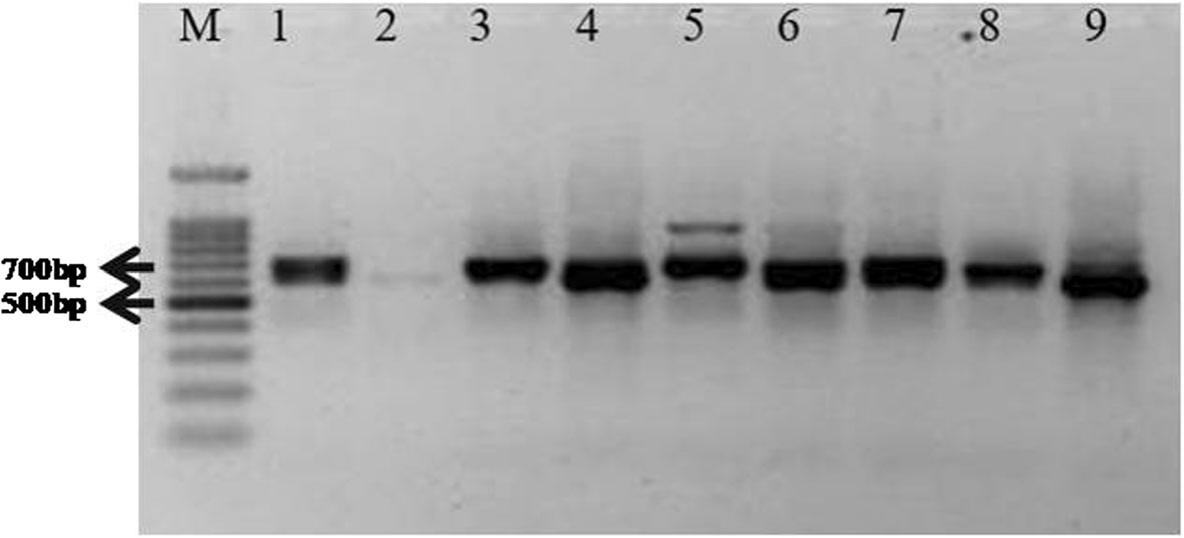
Comparative amplification of rice ITS using universal primer. Rice varieties Rajendra Sweta, HUR917, and TKM13 were used for the assay. M: 100 bp marker (Promega); Lane 1: TKM 13_Wang 2016; Lane 2: HUR 917_Wang 2016; Lane 3: TKM 13_Wang 1993; Lane 4: HUR 917_Wang 1993; Lane 5: TKM 13_Satya 2013; Lane 6: HUR 917_Satya 2013; Lane 7: TKM 13_rHTTP method; Lane 8: HUR 917_rHTTP method; Lane 9: Rajendra sweta_rHTTP method

Comparative analysis of RAPD profiles of two rice varieties was done and amplification of ITS region following the different protocols detailed in methods revealed positive results with excellent resolution with newly developed rHTTP method and protocol described by Satya et al. (Figs. 6 and 7). Protocols developed by Steiner et al. (1995) and Wang et al. (2016) failed to yield any amplification product or RAPD profiles. In case of the method described by Wang et al. (2016), very faint amplicon resulted in case of rice variety HUR 917 (Fig. 7).

## Discussion

In the present study, we have developed a simple, rapid, inexpensive protocol to prepare DNA template for PCR assays. In the rHTTP method development, the two most important factors were the concentration of SDS and the dilution factor. It is a well-known fact that SDS, an anionic detergent is inhibitory for Taq Polymerases [20]. In the present study also it was observed that bacterial DNA containing a final concentration of 0.0125% SDS in PCR reactions did not produce any amplification but amplification was obtained in bacterial DNA devoid of SDS. SDS has been listed among the chemicals having low concern regarding its environmental risks according to the United States Environmental Protection Agency and other relevant studies [21]. No amplification in case of crude extract from rHTTP lysates could be attributed to high concentration of both SDS (0.125%) and DNA (768.3 ng/μl). However, 10X and 20X diluted lysates for which the final concentration of SDS were 0.0062 and 0.0031% respectively showed strong amplifications. Despite no variation in amplification patterns between 10X and 20X diluted lysates, the latter was chosen to completely rule out any chance of presence of PCR inhibitors. Earlier also, Satya et al. (2013) emphasized that the dilution factor was the most important factor for success of such direct PCR methods [15].

Although, available literature showed a number of reports [12–15, 18] for rapid DNA template preparation for direct PCR assay, their use is still very limited. It has been reported that reliability, reproducibility of such techniques, shelf life of the extracts, quality and quantity of the DNA extracted etc. might have affected the utility and popularity of such techniques [15]. However, most of the techniques still require a good amount of instrumentation and tissue grinding which is probably the most monotonous act of any molecular biology laboratory. In many of these methods, liquid nitrogen or lyophilization is required which further add to the costs involved in these techniques. The rHTTP method developed in this study requires almost no technical expertise to execute and it requires no tissue grinding, vortexing or centrifugation. One most striking feature of this method is that it can be used outside any laboratory and without any sophisticated equipment. The only thing required is a means to boil water. Hence, the rHTTP method can be used for on-field experiments like molecular diagnostics, varietal identification, plant genotyping etc. Wang et al. (2016) reported a NaOH based modified DNA extraction protocol which they used for identification of transgenic lines in China [14]. However, the method reported by Wang et al. (2016) did not produce good results when we compared it to our method along with few other methods. The rHTTP method in conjunction with isothermal amplification technology can be a revolution in case of molecular diagnostics.

rHTTP method is high throughput method which can bring down the average sample processing time to few seconds. For example, if we want to process 100 samples at a time, the time required will be 10 min plus the time required for dispensing the lysis buffer and time required for making dilutions. However, for the same 100 samples, the other methods as well as any standard kit will take hours as the samples have to be ground or vortexed or centrifuged individually or in a series of very limited numbers. We have made a comparison with five other methods of rapid DNA template preparation and few commercially available kits for plant DNA extraction (Tables 2 and 3) with special reference to time, cost and instrumentation. Thermo Fisher Scientific Phire Plant Direct PCR Kit recommends the use of young leaves and essentially requires Phire Hot Start II DNA Polymerase which significantly increases the cost per sample and the use of commonly available Taq polymerases is not applicable in this case. Its dilution protocol involves tissue grinding as well as spin centrifuge which increases time and effort. Among the commercial kits available globally, only the QuickExtract™ DNA Plant DNA Extraction Solution marketed by Lucigen, USA is comparable to rHTTP method in terms of time and requirement of instrumentation. However, the sample needs to be heated at two different temperatures in case of this kit which in turn necessitates the use of a thermal cycler or a heating device with instant temperature controller. On the other hand, rHTTP method can be executed using boiling water which eliminates the temperature controlling step altogether. Nonetheless, the cost for template preparation per sample for this kit is almost 10 times higher as compared to rHTTP method. In majority of the commercially available kits, the cost per preparation ranges between $ 3–4.5 (INR 200–300) while the same is approximately $ 0.06 (INR 4.5; Additional file 4: Table S3) per preparation i.e., almost 50–75 times lesser for rHTTP method (Table 3). However, we believe that the rHTTP method is more suitable for PCR based studies where the quality of the DNA can be sacrificed to certain extent to save a considerable amount of time and cost. For identification, diagnostics and genotyping methods using PCR will find the major applications of the present method. The purity of the template DNA prepared using rHTTP method suggests that it may not be suitable for restriction analyses or genome sequencings. The DNA templates prepared with rHTTP method has an excellent storability. At − 20 °C, templates can be stored for 1 month while at room temperature it could be stored for at least 3 days.

**Table 2:** Comparison of rHTTP method with other reported methods of quick plant DNA extraction

	Rapid One Step Extraction (ROSE) method (Steiner et al., 1995)	Sucrose Prep Method (Berendzen et al. 2005)	NaOH Method (Wang et al., 1993)	NaOH Method (Satya et al. 2013)	NaOH Method (Wang et al., 2015)	rHTTP method (Present study)
Type and form of plant material	Lyophilized plant tissue	Fresh tissue	Young tissue	Fresh tissue	Fresh tissue	Fresh/old tissue
Liquid nitrogen	No	Yes/Ice	No	No	No	No
Extraction/lysis buffer	Tris-HCl, EDTA, Sodium Sarkosyl sulfate, PVPP	Tris-HCl, NaCl, Sucrose	NaOH, Tris	NaOH, Tris-EDTA	NaOH	SDS
Instrumentation	Lyophilizer, Shaker, hybridization Oven/ Water bath	Water Bath/Thermal cycler, Centifuge, grinding assembly/Mortar pestle	Grinding assembly/ Mortar-pestle	Grinding assembly/ Mortar-pestle, Centrifuge	Grinding assembly/ Mortar-pestle/ centrifuge	Thermal cycler/hot plate/any means to boil water
Duration to make templates for PCR (as per original publication)	~ 45 min after lyophilization	~ 15 min	Should not be more than 10 min	10–12 min	5 min	10 min
Actual time required to process five samples at a time	ND	59 min (11.8 min per sample) *For larger number of samples time will increase proportionately due to grinding	31 min (6.2 min per sample) *For larger number of samples time will increase proportionately due to grinding	65 min (13 min per sample) *For larger number of samples time will increase proportionately due to grinding	29 min (5.8 min per sample) *For larger number of samples time will increase proportionately due to grinding	12.5 min (2.5 min per sample) *For larger number of samples, the time will remain almost same as there is no grinding or maceration. The time will be 10 min plus time required for pipetting 100 μl lysis buffer

**Table 3:** Comparison of rHTTP method with few commercially available kits

Kits/methods	Time required per sample	Instrumentation	Cost
DNeasy Plant Mini Kit (Qiagen)	~ 60 min	Grinding assembly/ Mortar-pestle, Centrifuge, vortex (6 centrifugation steps)	INR 282/− per prep
Synergy™ 2.0 (OPS Diagnostics)	43 min	Centrifuge, vortex (5 centrifugation steps)	INR 153/− per prep
Plant DNA isolation reagent (Takara)	30 min.	Centrifuge, vortex (3 centrifugation steps)	INR 138/− per prep
Phire Plant Direct PCR Kit (Thermo Fisher Scientific)	–	Thermal cycler (sample lysis and PCR done in a single tube). Crushing and spinning required in Dilution protocol	INR 51/− per prep
QuickExtract™ Plant DNA extraction solution (Lucigen)	8 min	Heating device with temperature controller (it requires incubation at two different temperatures)	INR 40/− per prep
rHTTP method	10 min	Thermal cycler/ heating device/ any means to boil water	INR 4.5/− per prep

rHTTP method can be used for a variety of plant species belonging to Poaceae, Fabaceae, and Brassicaceae which encompass majority of the agriculturally important crops and most of the marker assisted breeding programs are targeted to these crops. Both random markers like RAPD and targeted amplification of Internal Transcribed Spacer (ITS) were obtained for all the plant species used. In order to further validate the applicability of the method, SSR markers were amplified in rice following the rHTTP method. This method can be very helpful in molecular breeding programs, plant genotyping where huge number of varieties or lines need to be screened using a large number of PCR based markers.

## Conclusions

The rHTTP holds a number of advantages over most of the above mentioned protocols- (i) No requirement of ice, liquid nitrogen or lyophilization, (ii) No tissue grinding, (iii) No centrifugation, (iv) No harmful chemicals, (v) This is high throughput method. Unlike, other methods, average sample processing time goes down with increasing number of samples (vi) Equally efficient for fresh as well as old tissue, (vii) It can work for a wide range of plant species, (viii) Requires almost no technical expertise, (ix) A single prep can be used for more than 500 PCR reactions (40 μl), (ix) The lysates have a month long shelf life when stored at − 20 °C, and (x) Cost per preparation is very low as compared to any other direct PCR method or commercially available kit. Hence, the rHTTP method described here is simpler, cheaper and more rapid as compared to other direct PCR systems reported till date and will be very useful in plant biotechnology projects having versatile PCR based applications.

## Methods

### Plant samples

A total of 21 different plant species belonging to 12 different families viz. Amaryllidaceae, Nyctaginaceae, Asteraceae, Apocynaceae, Solanaceae, Commelinaceae, Arecaceae, Fabaceae, Capparaceae, Verbenas, Poaceae, and Brassicaceae collected from different parts of India were used in the study (Refer Additional file 4: Table S1 for details of samples). All samples were collected in polypropylene bags and stored at 4 °C prior to use.

### Protocol development and optimization

A linear gradient of 0.5 to 5% (w/v with an interval of 0.5%) molecular biology grade Sodium Dodecyl Sulfate (HiMedia, India) prepared in MiliQ water was tested as lysis buffer. Final concentration of SDS was selected on the basis of DNA quantity and quality in the lysate prepared with two leaf discs. Initially, the lysis was done at 99 °C for 20 min. Varying amount of tissue was used for optimizing the amount of start sample as it affects the quantity and quality of DNA template to be prepared. For this, variable number of leaf discs (~ 0.5 cm, diameter) viz. one disc (~ 1.6 mg) and two discs (~ 3.2 mg) were used for lysis as mentioned above. After optimizing the concentration of SDS and amount of tissue to be lysed, the time for lysis was further optimized using two time periods for lysis viz. 10 and 20 min at 99 °C.

For protocol optimization, leaves of the rice variety HUR917 were used. For lysis, the leaf discs were placed in a 200 μl PCR tubes (Tarsons, India) and 100 μl of lysis buffer was added to it and heated in a thermal cycler at 99 °C (PeqStar, VWR International Ltd., UK). All lysis and spectrophotometric observations were carried out in five replicates.

### Initial assessment of DNA template quality and quantity

DNA concentration and purity (A_260_/A_280_) was determined using ScanDrop spectrophotopmeter (Analytik Jena AG, Germany).

### PCR assay

For optimization of PCR amplification, crude lysate, 10X, and 20X dilutions of the lysates were used as template. Dilutions were prepared in MiliQ water. GoTaq® Green Mastermix (Promega, USA) was used throughout the experiments. An aliquot of 2.5 μl of the template was used in two reaction volumes viz. 20 and 40 μl. For 40 μl reaction volume, 10X dilutions as well as 20X dilutions were used and for 20 μl reaction volume, only crude extract was used. With an assumption that lysates will have a significant amount of PCR inhibitors, all PCR reactions were made in two sets, one with Bovine Serum Albumin (Promega, USA) as a PCR enhancer with a final concentration of 0.01 μg/ml and another without BSA. For comparison, bacterial genomic DNA suspended in 0.1% SDS and TE buffer was also used for RAPD in a 20 μl reaction volume.

### Primers and PCR conditions

For optimization of initial PCR conditions, RAPD primers listed in Additional file 4: Table S2 were used. The following conditions were used-Initial denaturation: 94 °C for 45 s, ten cycles of (denaturation: 94 °C for 45 s, primer annealing: 45 °C for 60 s, primer extension: 72 °C for 2 min), 30 cycles of (denaturation: 94 °C for 45 s, primer annealing: 45 °C for 45 s, primer extension: 72 °C for 2 min), final primer extension: 72 °C for 10 min. For comparison with other methods, both RAPD primers and universal plant internal transcribed spacer (ITS) specific primers were used (Additional file 4: Table S2) [22]. For amplification of plant specific ITS region, following PCR conditions were used- Initial denaturation: 94 °C for 4 min, 34 cycles of (denaturation: 94 °C for 30 s, primer annealing: 58 °C for 40 s, primer extension: 72 °C for 60 s), final primer extension: 72 °C for 10 min.

### Effectiveness of the optimized method

To check the effectiveness of the optimized method, it was tested on 11 different rice varieties, wheat, pigeonpea, mustard, soybean, pea, tomato, maize, march lilly, bougainvillea, Indian blanket flower, nerium, petunia, purple pirouette petunia, moses-in-the-cradle, golden cane palm, duranta, periwinkle, chrysanthemum, *Dipterygium glaucum* and *Crotaleria burhia*. SSR markers specific for rice viz. RM18398 and RM26108 (Additional file 4: Table S2) were used for amplification with rHTTP templates [23]. Further the effectiveness of the protocol was tested with the leaves of 2 months old leaf tissues of rice varieties PR108, PR111 and two other local varieties collected from Punjab, India. The integrity of the template DNA prepared using optimized protocol during storage was studied by keeping the lysates at − 20 °C or at room temperature. The lysates were stored at − 20 °C for 30 days and PCR amplification was checked at an interval of 10 days. Likewise the lysates were kept at room temperature for a week and checked through PCR amplification at an interval of 3 days.

### Testing of the method using boiling water

After optimization of all the parameters viz. SDS concentration, amount of tissue and time, the protocol was further tested for its execution using boiling water instead of thermal cycler so that it can be performed even outside the laboratory. For this, water was boiled on hot plate (Impact Icon Instruments Company, India) and temperature of the water was monitored with a digital thermometer (Frontier Multi- Thermometer, Model: ST-9283, India). The PCR tubes containing leaf discs and lysis buffer were put into a beaker containing boiling water. Lysis was carried out for two time periods viz. Ten and 20 min as the surface temperature of the boiling water ranged from (100–102 °C).

### Comparison with other similar methods

Four quick plant DNA extraction protocols previously reported by- (i) Wang et al. (1993), (ii) Wang et al.(2016), (iii) Satya et al. (2013), and (iv) Steiner et al. (1995) were compared with the optimized protocol in terms of amplification profiles using RAPD and ITS specific primers. A comparison in terms of time, cost and instrumentation was also made with few popular commercial kits for plant DNA isolation.

## Supplementary information

**Additional file 1: Figure S1.** RAPD profile of 11 different varieties of rice using OPB06 primer. M: 100 bp marker (Promega); Lane 1: TKM 13; Lane 2: Rajendra Sweta; Lane 3: HUR 105; Lane 4: Improved Pusa Basmati 1; Lane 5: GM 96; Lane 6: GM 99; Lane 7: GM 137; Lane 8: GM 113; Lane 9: Pusa Basmati 1121; Lane 10: PR 115; Lane 11: PR111.

**Additional file 2: Figure S2.** RAPD profile (with OPB07) of four different rice using 2 months old leaf tissue. Lane 1: PR111; Lane 2: PR108; Lane 3: Local variety 1; Lane 4: Local variety 2.

**Additional file 3: Figure S3.** Gel photograph showing the results of ITS amplification using 3 days old template DNA of rice and wheat stored at room temperature. M: 100 bp marker (Promega); Lane 1: Wheat (var. HD2967); Lane 2: Rice (var. HUR917)

**Additional file 4: Table S1.** Details of plant samples used in the study. **Table S2.** Primers (RAPD, ITS, and SSR) used in the study. **Table S3.** Calculation of cost per preparation for rHTTP method.

## Data Availability

All data generated or analysed during this study are included in this published article.

## Abbreviations

BSA: Bovine Serum Albumin
CRIJAF: Central Research Institute for Jute and Allied Fibers
DNA: Deoxyribonucleic acid
ICAR: Indian Council of Agricultural Research
IISS: Indian Institute of Seed Science
ITS: Internal Transcribed Spacer
LAMP: Loop Mediated Isothermal Amplification
NBAIM: National Bureau of Agriculturally Important Microorganisms
PCR: Polymerase Chain Reaction
RAPD: Random Amplified Polymorphic DNA
rHTTP: Rapid High Throughput Template Preparation
ROSE: Rapid One-step Extraction
SDS: Sodium Dodecyl Sulphate
SE: Standard Error
TE: Tris EDTA
