# Supplementary figures and images for: Rapid high throughput template preparation (rHTTP) method: a novel cost effective method of direct PCR for a wide range of plants
Source: BMC Biotechnol. 2019 Oct 26;19:69. doi: 10.1186/s12896-019-0560-4 (PMC6815007; doi:10.1186/s12896-019-0560-4)

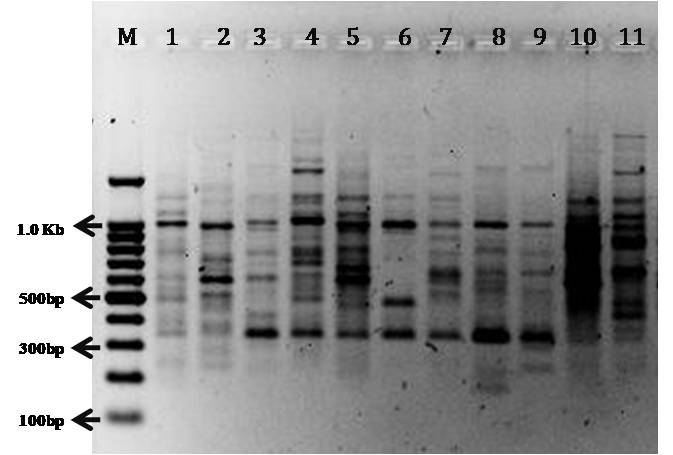

Supplement: Supplementary file 1 — Additional file 1: Figure S1. RAPD profile of 11 different varieties of rice using OPB06 primer. M: 100 bp marker (Promega); Lane 1: TKM 13; Lane 2: Rajendra Sweta; Lane 3: HUR 105; Lane 4: Improved Pusa Basmati 1; Lane 5: GM 96; Lane 6: GM 99; Lane 7: GM 137; Lane 8: GM 113; Lane 9: Pusa Basmati 1121; Lane 10: PR 115; Lane 11: PR111. [file 12896_2019_560_MOESM1_ESM.jpg]

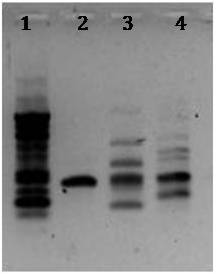

Supplement: Supplementary file 2 — Additional file 2: Figure S2. RAPD profile (with OPB07) of four different rice using 2 months old leaf tissue. Lane 1: PR111; Lane 2: PR108; Lane 3: Local variety 1; Lane 4: Local variety 2. [file 12896_2019_560_MOESM2_ESM.jpg]

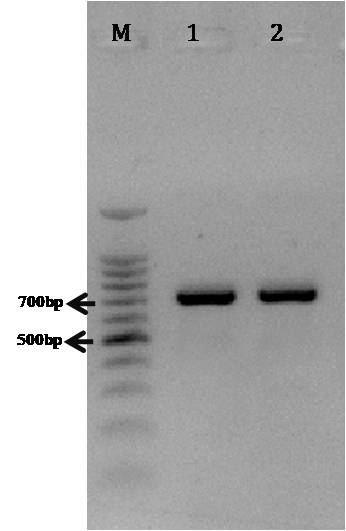

Supplement: Supplementary file 3 — Additional file 3: Figure S3. Gel photograph showing the results of ITS amplification using 3 days old template DNA of rice and wheat stored at room temperature. M: 100 bp marker (Promega); Lane 1: Wheat (var. HD2967); Lane 2: Rice (var. HUR917) [file 12896_2019_560_MOESM3_ESM.jpg]
