# Supplementary material for: Rapid high throughput template preparation (rHTTP) method: a novel cost effective method of direct PCR for a wide range of plants
Source: BMC Biotechnol. 2019 Oct 26;19:69. doi: 10.1186/s12896-019-0560-4 (PMC6815007; doi:10.1186/s12896-019-0560-4)
Supplement: Supplementary file 4 — Additional file 4: Table S1. Details of plant samples used in the study. Table S2. Primers (RAPD, ITS, and SSR) used in the study. Table S3. Calculation of cost per preparation for rHTTP method. [file 12896_2019_560_MOESM4_ESM.docx]

**Table S1**: Details of plant samples used in the study

| Sl. no | Plant sample | Variety/ Germplasm | Source | Plant part used |
| --- | --- | --- | --- | --- |
| 1 | Rice | TKM 13 | IISR fields, Mau, India | Leaf/ Leaf sheath |
| 2 | Rice | Rajendra Sweta | IISR fields, Mau, India | Leaf/ Leaf sheath |
| 3 | Rice | SHIATS 1 | IISR fields, Mau, India | Leaf/ Leaf sheath |
| 4 | Rice | Improved Pusa Basmati 1 | IISR fields, Mau, India | Leaf/ Leaf sheath |
| 5 | Rice | GM 96 | IISR fields, Mau, India | Leaf/ Leaf sheath |
| 6 | Rice | GM 99 | IISR fields, Mau, India | Leaf/ Leaf sheath |
| 7 | Rice | GM 137 | IISR fields, Mau, India | Leaf/ Leaf sheath |
| 8 | Rice | GM 113 | IISR fields, Mau, India | Leaf/ Leaf sheath |
| 9 | Rice | HUR 105 | IISR fields, Mau, India | Leaf/ Leaf sheath |
| 10 | Rice | HUR 917 | IISR fields, Mau, India | Leaf/ Leaf sheath |
| 11 | Rice | Pusa Basmati 1121 | IISR fields, Mau, India | Leaf/ Leaf sheath |
| 12 | Rice | KS Dev 12 | IISR fields, Mau, India | Leaf/ Leaf sheath |
| 13 | Rice | MTU 5204 | IISR fields, Mau, India | Leaf/ Leaf sheath |
| 14 | Rice | PR111 | Amritsar, India | Leaf/ Leaf sheath |
| 15 | Rice | PR108 | Amritsar, India | Leaf/ Leaf sheath |
| 16 | Rice | Local variety 1 | Amritsar, India | Leaf/ Leaf sheath |
| 17 | Rice | Local variety 2 | Sirhand, India | Leaf/ Leaf sheath |
| 18 | Mustard | Pusa bold | IISR fields, Mau, India | Leaf |
| 19 | Soybean | P S 1347 | IISR fields, Mau, India | Leaf |
| 20 | Pigeonpea | Bahar | IISR fields, Mau, India | Leaf |
| 21 | Wheat | HD2967 | IISR fields, Mau, India | Leaf |
| 22 | Pea | Prakash | IISR fields, Mau, India | Leaf |
| 23 | Maize | - | IISR fields, Mau, India | Leaf |
| 24 | Tomato | - | IISR fields, Mau, India | Leaf |
| 25 | Desert xerophyte (*Dipterygium glaucum*) | - | Jaiselmer, India | Stem |
| 26 | Desert xerophyte (*Crotalaria burhia*) | - | Jaiselmer, India | Stem |
| 27 | March Lilly (*Amaryllis belladonna*) | - | NBAIM garden, Mau, India | Leaf |
| 28 | Bougainvillea (*Bougainvillea glabra*) | - | NBAIM garden, Mau, India | Leaf |
| 29 | Indian Blanket flower (*Gaillardia pulchella*) | - | NBAIM garden, Mau, India | Leaf |
| 30 | Nerium (*Nerium oleander*) | - | NBAIM garden, Mau, India | Leaf |
| 31 | Petunia (*Petunia* sp.) | - | NBAIM garden, Mau, India | Leaf |
| 32 | Purple Pirouette Petunia (*Petunia* sp.) | - | NBAIM garden, Mau, India | Leaf |
| 33 | Moses-in-the cradle (*Rheo discolor*) | - | NBAIM garden, Mau, India | Leaf |
| 34 | Golden cane palm (*Dypsis lutescens*) | - | NBAIM garden, Mau, India | Leaf |
| 35 | Duranta (*Duranta plumieri*) | - | NBAIM garden, Mau, India | Leaf |
| 36 | Periwinkle (*Catharanthus roseus*) | - | NBAIM garden, Mau, India | Leaf |
| 37 | Chrysanthemum (*Chrysanthemum* sp.) | - | NBAIM garden, Mau, India | Leaf |

**Table S2:** Primers (RAPD, ITS, and SSR) used in the study

| Sl. no. | Primer name | Sequence |
| --- | --- | --- |
| 1 | OPB06 | TGCTCTGCCC |
| 2 | OPB07 | GGTGACGCAG |
| 3 | ITS (forward) | GGAAGKARAAGTCGTAACAAGG |
| 4  5  6  7  8 | ITS (reverse)  RM18398F  RM18398R  RM26108F  RM26108R | RGTTTCTTTTCCTCCGCTTA  CCCTTTGCTCTGAATCTGATTACC  GGCTCAAAGTAGTGCTCCATCC  TTGTGCCTGTGTCAGAAGAACTAGG  GCAAAGGTAGCTAACACACATACGG |

**Table S3:** Calculation of cost per preparation for rHTTP method

| **Material** | **Used for** | **Cost (INR) per preparation** |
| --- | --- | --- |
| PCR tube- 0.2 ml (Tarsons, India) | Lysis | 2.41 |
| Micro centrifuge tube -1.5 ml (Tarsons) | Dilution of lysate | 0.80 |
| SDS (HiMedia, India) | Lysis | 0.011 |
| Micro Tips- 0.2 ml (Tarsons, India) | Dispensing lysis buffer | 1.22 |
| **Total** | | **4.441=~4.50** |
